# Predictors of Blunt Thoracic Aortic Injury Requiring TEVAR in Patients with Left-Sided Hemothorax: Implications for Chest Drainage and Early CTA Assessment

**DOI:** 10.3390/jcm15114183

**Published:** 2026-05-28

**Authors:** Giovanni Zambello, Alessandro Bonis, Riccardo Amatucci, Birgit Feil, Luiz Felippe Milazzo, Marco Damiano Pipitone, Filippo Gorgatti, Giovanni Coppi, Reinhold Perkmann, Francesco Zaraca

**Affiliations:** 1 Department of Vascular and Thoracic Surgery, Provincial Hospital of Bolzano (SABES-ASDAA), Teaching Hospital of Paracelsus Medical University, 39100 Bolzano-Bozen, Italy; birgit.feil@sabes.it (B.F.); luizfelippe.milazzo@sabes.it (L.F.M.); marcodamiano.pipitone@sabes.it (M.D.P.); filippo.gorgatti@sabes.it (F.G.); giovanni.coppi@sabes.it (G.C.); reinhold.perkmann@sabes.it (R.P.); francesco.zaraca@sabes.it (F.Z.); 2 Unit of Thoracic Surgery, Department of Cardiac, Thoracic, Vascular Sciences and Public Health, University of Padua, 35128 Padua, Italy; alessandro.bonis@aopd.veneto.it; 3 Thoracic Surgery Unit, Department of Surgical Sciences, Santa Maria della Misericordia Hospital, University of Perugia Medical School, 06129 Perugia, Italy; riccardo.amatucci94@gmail.com

**Keywords:** blunt thoracic aortic injury, TEVAR, hemothorax, trauma

## Abstract

**Background:** Blunt thoracic aortic injury (BTAI) is an uncommon but life-threatening consequence of blunt thoracic trauma. Left-sided hemothorax is frequently identified during initial evaluation and typically prompts early chest drainage. However, when an unrecognized BTAI is present, pleural decompression may precipitate hemodynamic instability. This study aimed to identify early predictors of BTAI requiring thoracic endovascular aortic repair (TEVAR) in patients presenting with left-sided hemothorax. **Methods:** We conducted a single-center retrospective cohort study including consecutive trauma patients aged ≥ 16 years with radiologically confirmed left-sided hemothorax between 2015 and 2025. Patients were stratified according to the need for TEVAR. Clinical, laboratory, and radiological variables available at emergency department admission were analyzed. Independent predictors of BTAI requiring TEVAR were identified using multivariable logistic regression. **Results:** Among 146 included patients, 27 (18%) underwent TEVAR for confirmed BTAI. Patients requiring TEVAR were generally younger and more frequently involved in high-energy trauma. Independent predictors of TEVAR included high-energy mechanism (*p* = 0.048), lower admission hemoglobin (*p* = 0.007), presence of extra-thoracic fractures (*p* < 0.001), and a higher number of right-sided rib fractures (*p* = 0.018). The volume of left-sided hemothorax was not independently associated with BTAI. The model demonstrated strong discriminative ability (AUC = 0.926). **Conclusions:** In trauma patients with left-sided hemothorax, BTAI requiring TEVAR may occur even in the presence of minimal pleural effusion. Readily available admission parameters may help identify patients who could benefit from a CT angiography-first approach rather than routine early chest drainage, except in cases of immediate life-threatening pleural compromise.

## 1. Introduction

Thoracic trauma is the second most common type of traumatic injury, accounting for approximately 35% of trauma-related deaths and representing a leading cause of mortality worldwide [1]. Blunt chest trauma constitutes about 15% of all trauma cases, and injury to the thoracic aorta is one of its most lethal complications [2,3]. Although the incidence of blunt thoracic aortic injury (BTAI) is relatively low, mortality remains extremely high, with up to 80% of patients dying before reaching hospital and only a small proportion surviving long enough to receive definitive treatment [4]. Early diagnosis and endovascular management, rather than emergent open surgery, have been shown to significantly improve survival among hospitalized patients [5,6,7]. Autopsy studies have demonstrated that hemothorax is present in the majority of patients with fatal BTAI (86.3% versus 55.7% in those without aortic injury), highlighting a strong association between these conditions in lethal scenarios [8]. Hemothorax is a common consequence of blunt chest trauma and is typically managed with early pleural drainage, in accordance with Advanced Trauma Life Support (ATLS) principles, which prioritize airway and breathing over circulation during the primary survey [9,10]. However, in the presence of an unrecognized BTAI, chest tube placement may precipitate sudden hemodynamic collapse by releasing a contained mediastinal hemorrhage. This phenomenon has been consistently reported in case reports and small case series, particularly in patients with left-sided hemothorax [11,12,13,14,15]. Despite this risk, there are currently no practical criteria to identify patients with left-sided hemothorax who are at high risk of clinically significant aortic injury during the initial emergency department assessment. Existing evidence largely focuses on imaging-based diagnosis, while early clinical and radiological indicators that may raise suspicion before definitive imaging remain poorly defined [16,17]. In particular, the role of hemothorax volume and associated injury patterns in predicting the need for thoracic endovascular aortic repair (TEVAR) is still debated. This study aimed to evaluate the prevalence of BTAI in patients presenting with left-sided hemothorax after blunt trauma and to identify admission clinical, laboratory, and radiological predictors associated with BTAI requiring TEVAR. Ultimately, the goal was to improve early clinical decision-making and optimize the timing of chest drainage in the acute trauma setting.

## 2. Materials and Methods

This retrospective observational cohort study was conducted at Bolzano Central Hospital (Bolzano, Italy), which is classified as a trauma center under the Italian emergency care system (DEA level II). The study period ranged from 1 January 2015 to 31 December 2025. Although the management of BTAI evolved over time, the core diagnostic pathway and the indications for TEVAR remained consistent at our institution throughout the study period. In detail, indications for this procedure were based on the European classification for BTAI and the recommendations of the Society for Vascular Surgery [18]. All consecutive patients admitted to the Department of Thoracic and Vascular Surgery following blunt trauma were screened. According to institutional protocol, patients with complex thoracic trauma patterns are routinely admitted to our department, either directly from the emergency department (ED) or after intensive care unit (ICU) management, even in the absence of suspected major vascular injury. Therefore, the study population reflects the case-mix of patients with moderate-to-severe thoracic trauma evaluated by a specialized thoracic team.

**Figure 1:**
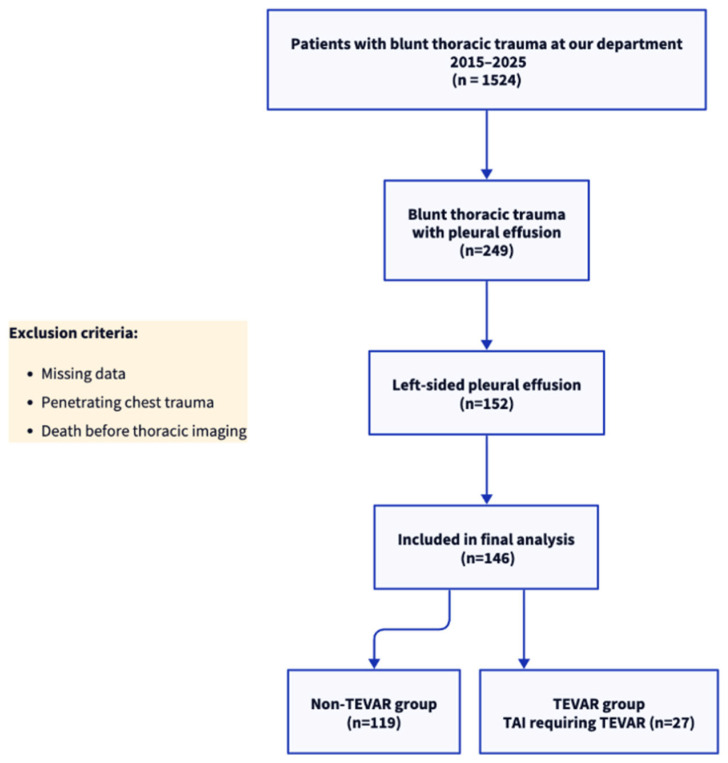
Flowchart of patient selection and study cohort formation.

Inclusion criteria were age ≥ 16 years, blunt trauma mechanism, and radiological evidence of left-sided hemothorax on chest X-ray and/or CT scan at hospital admission. Exclusion criteria were penetrating chest trauma, death before thoracic imaging, inter-hospital transfer with incomplete imaging or outcome data, and isolated right-sided hemothorax without left hemithorax involvement. Patients were selected based on predefined clinical and radiological criteria (Figure 1).

### 2.1. Study Groups

Patients were divided into two groups according to whether they required TEVAR during hospitalization. The TEVAR group comprised patients with left-sided hemothorax and imaging evidence of thoracic aortic injury (BTAI) requiring endovascular treatment. The non-TEVAR group included patients with left-sided hemothorax without imaging evidence of BTAI. For the purposes of this study, BTAI was defined as disruption of the aortic wall on computed tomography (CT) and/or CT angiography, including extravascular blood collection and/or mediastinal hematoma [19]. Injury severity was further classified according to the grading system proposed by Azizzadeh et al. [20].

The presence of left-sided hemothorax was used as an inclusion criterion and was not part of the diagnostic definition of BTAI. Patients with traumatic aortic injury limited to the intimal layer and without radiological evidence of hemothorax were excluded.

### 2.2. Data Collection and Imaging Assessment

This retrospective observational study consisted of a retrospective analysis of anonymized routinely collected clinical data from patients managed at Bolzano Hospital. Ethical approval was granted by the local Ethics Committee at Bolzano Hospital in accordance with the Declaration of Helsinki (Approval No. 140/2020; Protocol Code: 0187299-BZ; approved on 18 December 2020). All data were anonymized and analyzed in accordance with institutional data protection policies and the General Data Protection Regulation (GDPR; EU Regulation 2016/679). Data were extracted from electronic medical records and radiological archives, including demographic characteristics, trauma mechanism, clinical presentation, imaging findings, treatment strategy, and clinical outcomes.

**Figure 2:**
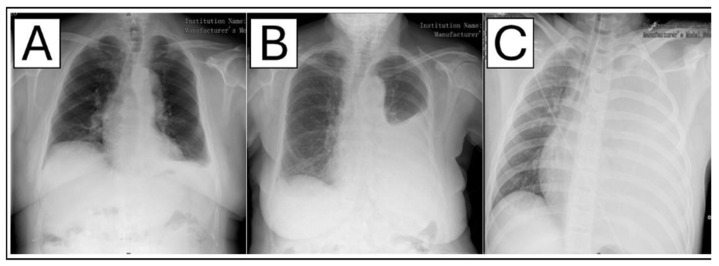
Semi-quantitative classification of left-sided hemothorax on chest radiography at admission. Representative chest X-rays showing (**A**) small left-sided hemothorax, defined as blunting of the left costophrenic angle; (**B**) moderate left-sided hemothorax, extending to approximately half of the left hemithorax and partially obscuring the cardiac silhouette; and (**C**) massive left-sided hemothorax, with near-complete opacification of the left hemithorax.

The extent of left-sided hemothorax was semi-quantitatively assessed at hospital admission based on chest radiography (chest X-ray or initial scout image before CT), which represented the primary imaging modality during the initial trauma evaluation. Hemothorax was classified as small (limited to costophrenic angle blunting), moderate (extending to approximately half of the hemithorax, covering the cardiac silhouette), or massive (near-complete opacification of the hemithorax) (Figure 2).

These categories correspond to approximate blood volumes of <500 mL, 500–1000 mL, and >1000 mL, respectively, as commonly adopted in clinical trauma practice [21]. When CT was available, the radiographic classification was confirmed but not reclassified, in order to reflect the information available at the time of initial clinical decision-making. The management strategy was categorized as CT-first, drain-first, or no drain during the initial assessment. This choice was not protocolized and may have reflected clinician judgment.

### 2.3. Outcomes

The primary outcome was the occurrence of BTAI requiring TEVAR in patients presenting with left-sided hemothorax following blunt trauma.

Secondary outcomes were the identification of clinical, laboratory, and radiological patterns associated with BTAI and comparison between the TEVAR group and patients managed without TEVAR. The ultimate aim was to identify combinations of predictors that may represent red flags to avoid a drain-first approach in patients with left-sided hemothorax after blunt thoracic trauma.

### 2.4. Statistical Analysis

Continuous variables are reported as median and interquartile range, and categorical variables as absolute numbers and percentages. Comparisons between groups were performed using appropriate non-parametric or categorical tests, as indicated.

A multivariable binomial logistic regression model was developed to identify predictors of TEVAR requirement. Clinical predictors were selected a priori based on availability at admission and a strategy prioritizing sensitivity over specificity, aiming to minimize inappropriate chest drain placement in patients with possible BTAI.

The following variables were included: (1) trauma mechanism (high- vs. low-energy), (2) admission hemoglobin level (g/dL), (3) number of right-sided rib fractures, and (4) presence of extra-thoracic fractures.

Trauma energy was classified according to established prehospital and trauma registry criteria. High-energy trauma included falls > 1 m, motor vehicle collisions with structural intrusion, motorcycle crashes, and pedestrian–vehicle impacts; all other mechanisms (e.g., ground-level falls ≤ 1 m or fewer than five stairs) were classified as low-energy trauma [22,23]. A *p*-value < 0.05 was considered statistically significant. Statistical analyses were performed using Jamovi software (The Jamovi Project, Sydney, Australia), version 2.6.45.

Given the limited number of outcome events, the multivariable model was intentionally restricted to a small set of clinically relevant predictors available at admission to reduce the risk of overfitting and improve model stability. Predictors were selected a priori based on clinical relevance and to minimize potential multicollinearity between variables. Model performance was estimated using 5-fold cross-validation. The mean discriminative performance, expressed as AUC, was 0.92 (SD 0.04), remaining consistent with the apparent model performance despite the limited sample size and number of events (RStudio, 2025.05.0 version, pROC package).

## 3. Results

### 3.1. Study Population and Baseline Characteristics

A total of 146 patients met the inclusion criteria. The cohort was mainly composed of males (73%), and the sample had a median age of 64 years (IQR: 50–80). High-energy trauma mechanisms accounted for 59% of cases, with road traffic collisions and falls representing the most frequent trauma dynamics. At hospital admission, 12% of patients presented with hemodynamic shock, and 7.2% required prehospital endotracheal intubation. Polytrauma CT during the initial assessment was performed in 43% of patients. Traumatic aortic disruption requiring TEVAR was identified in 27 patients (18%). Baseline demographic, clinical, and radiological characteristics of the entire cohort are summarized in Table 1.

**Figure 3:**
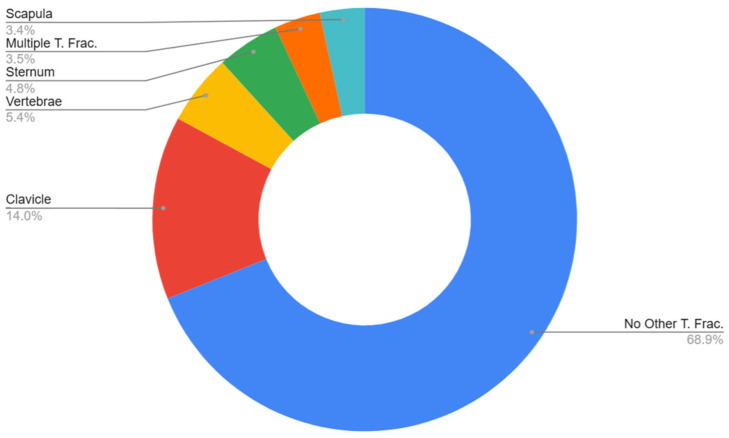
Proportion of the thoracic fractures beyond ribs involvement in the entire sample.

The distribution of thoracic fractures beyond rib involvement in the overall study population is shown in Figure 3.

### 3.2. Comparison Between TEVAR and Non-TEVAR Groups

**Figure 4:**
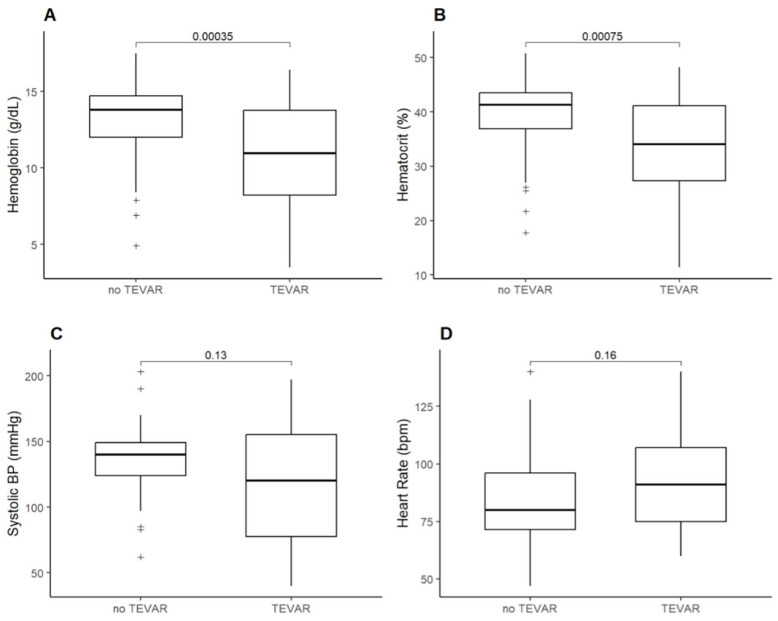
Clinical parameters at admission. Blood sample demonstrated a significant difference between the two groups, whereas blood pressure and heart rate were not significantly different. (**A**) Hemoglobin values; (**B**) Hematocrit values; (**C**) Systolic blood pressure (mmHg); (**D**) Heart rate (beats per minute).

Patients requiring TEVAR were significantly younger than those managed conservatively (median age: 50 vs. 68 years, *p* = 0.002) and were more frequently exposed to high-energy trauma mechanisms (89% vs. 52%, *p* < 0.001). TEVAR patients presented more often with hemodynamic instability at admission (70% vs. 2.5%, *p* < 0.001) and often required prehospital intubation (37% vs. 2.5%, *p* < 0.001). Laboratory findings showed significantly lower hemoglobin and hematocrit levels in the TEVAR group (Hb: 10.95 vs. 13.80 g/dL, *p* < 0.001; Hct: 34% vs. 41%, *p* < 0.001), whereas systolic blood pressure and heart rate at admission did not differ significantly between groups. These differences are illustrated in Figure 4.

From a radiological standpoint, mediastinal hematoma was strongly associated with TEVAR requirement (65% vs. 3.4%, *p* < 0.001). TEVAR patients more frequently exhibited bilateral rib fractures and associated extra thoracic fractures, while left-sided rib fracture burden alone was not discriminative. A detailed comparison between the two groups is reported in Table 2.

### 3.3. Predictors of BTAI

**Figure 5:**
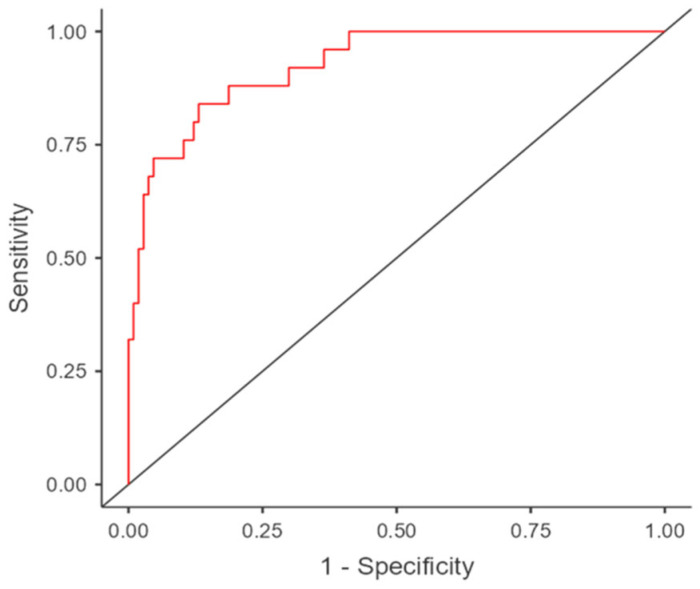
ROC curve for the logistic regression, with AUC of 0.926. The model shows high sensitivity but low specificity.

On binomial logistic regression analysis, high-energy trauma mechanism, lower hemoglobin levels at admission, presence of extra thoracic fractures, and a higher number of right-sided rib fractures were independently associated with BTAI requiring TEVAR. Conversely, the extent of left-sided rib fractures was not independently predictive. Results of the multivariable analysis are shown in Table 3. Receiver Operating Characteristic (ROC) curve analysis demonstrated satisfactory discriminative performance of the model in identifying patients requiring TEVAR (AUC = 0.926) (Figure 5).

Multivariable logistic regression demonstrated that high-energy trauma (OR 5.08, 95% CI 1.01–25.43, *p* = 0.048), extra thoracic fractures (OR 12.92, 95% CI 3.52–47.41, *p* < 0.001), increasing number of right rib fractures (OR 1.43, 95% CI 1.06–1.93, *p* = 0.018), and lower admission hemoglobin (OR 0.71, 95% CI 0.55–0.91, *p* = 0.007) were independently associated with risk of BTAI and TEVAR requirement in our experience (Table 3).

### 3.4. Management Strategy and Clinical Outcomes

During the initial trauma assessment, chest drainage was performed in 32 patients (22%). Among these, drainage was placed after chest X-ray or CT imaging in 13 cases (9%), for tension pneumothorax or minimal effusion in 9 cases (6.2%), and in the prehospital setting in 6 patients (4.1%). A CT-first strategy followed by direct operative management for BTAI was adopted in 4 patients (3%). Most patients (113/146, 77.4%) were managed without immediate chest drainage during the initial assessment. Delayed pleural evacuation was required in 13 patients (8.8%), including thoracentesis alone in 2 cases and chest tube placement in 11 patients. Among those undergoing chest drainage, the initial drained volume was <500 mL in most cases (85%), whereas volumes between 500 and 1000 mL and >1000 mL were observed in 11% and 3.5% of them, respectively.

Thoracic endovascular aortic repair was required in 27 patients (18%) of our highly selected sample. Additional surgical or endovascular procedures were infrequently required. Overall, in-hospital mortality was 3.5%, with 30-day and 90-day mortality rates of 0.7% each. No procedure-related mortality was observed following TEVAR (Table 4).

## 4. Discussion

In this retrospective cohort study of patients presenting with left-sided hemothorax after blunt trauma, two main findings emerged. Although still representing a small proportion of all thoracic traumas, BTAI requiring TEVAR was not uncommon in this selected cohort, affecting nearly one-fifth of our sample. Secondly, specific clinical and radiological patterns were consistently associated with BTAI, which may be recognizable during the initial trauma assessment and may act as red flags when admitting the patient to the Emergency Department. Notably, the main purpose of this study was not to provide a specific guideline, but to highlight peculiar characteristics that were consistently found in blunt traumas which required TEVAR due to BTAI.

The observed prevalence of traumatic aortic disruption in our cohort is markedly higher than that reported in unselected thoracic trauma populations, where BTAI has been estimated to occur in approximately 0.3 to 2% of cases [3,24]. This discrepancy likely reflects the highly selected nature of our study population, limited to patients with post-traumatic left-sided hemothorax, as well as the characteristics of a tertiary trauma center serving as a referral hub for high-energy trauma. Nevertheless, considering that we selected only left-sided patients with radiological evidence of hemothorax, the real prevalence of BTAI in our region remains in the range of statistics expected from a referral trauma center. In left-sided traumas, the presence of hemothorax appears to be a flag of severity rather than an isolated or benign finding, as previously demonstrated in recent studies [25]. Therefore, the extent of hemothorax should not be considered as a marker of severity, because in our patients requiring TEVAR, hemothorax was massive in only about 10% of cases. Conversely, this suggests that the absence of a large pleural effusion should not be considered reassuring, as small hemothorax can conceal severe vascular injury. Our findings are in line with current trauma guidelines, which emphasize that hemothorax volume alone should not dictate management decisions and that clinical stability remains the primary driver of intervention [26]. Accordingly, in our model the volume of blood effusion was intentionally omitted.

Importantly, guidelines support a selective approach to chest tube placement, reserving urgent drainage for massive or tension pneumothorax, mediastinal shifting hemothorax with clinical impairment, or physiological compromise. In such extreme cases, prioritizing chest drainage placement is a lifesaving maneuver, over the risk of BTAI and even death. At the same time, international recommendations acknowledge that small-volume hemothorax (approximately < 300–500 mL) may be safely observed in selected stable patients with well-reported traumatic dynamics [27,28]. In unstable patients with tension physiology or severe respiratory compromise, immediate pleural decompression should not be delayed by additional imaging. Nevertheless, clear criteria integrating hemothorax characteristics with the risk of major vascular injury remain lacking, particularly in blunt trauma. Our findings extend these recommendations by showing that traumatic aortic disruption may occur even in patients with minimal pleural effusion, supporting the prioritization of early computed tomography angiography (CTA) over routine drain-first strategies in selected high-risk patients, even in case of stable clinical parameters and a small left hemothorax.

Beyond the volume of pleural effusion, several other variables were independently associated with the need for TEVAR due to BTAI, including a high-energy trauma mechanism, hemoglobin levels below the normal range (12 g/dL), presence of extra thoracic fractures, and a higher number of right-sided rib fractures. These findings likely reflect the overall energy transfer and systemic impact of trauma rather than isolated thoracic damage as previously described in the literature [13]. Conversely, left-sided rib fracture burden alone was not predictive, supporting the idea that broader injury patterns may indicate a global transmission of the energy rather than a localized trauma, being more informative [29].

From a clinical perspective, these results have important implications for the timing of chest drainage. In patients with left-sided hemothorax and associated high-risk features, a drain-first strategy may expose patients to the risk of abrupt hemodynamic deterioration by disrupting a temporarily contained aortic hemorrhage [11,12,14,15]. In our cohort, a drain-first approach was frequently adopted during the initial assessment of patients with left-sided hemothorax, reflecting real-world trauma practice. The timing and clinical context of chest drainage may also influence hemodynamic stability in patients with occult BTAI and deserve further investigation.

Patients requiring TEVAR more frequently underwent prehospital chest drainage (Table 4), whereas patients not requiring TEVAR were more often managed with drainage after imaging or without drainage. Taken together, these findings suggest that, when clinically feasible, an imaging-first (CTA-first) approach may deserve consideration before routine drain-first strategies in selected high-risk patients. Meanwhile, in patients with no evidence of such predictors, normal guidelines and local protocols for the chest drain management may be followed, still monitoring the risk of unforeseen BTAI. This model demonstrated a satisfactory discrimination (AUC = 0.926) alongside reliance on simple and rapidly assessable predictors. Given the relatively small number of events, the model performance should be interpreted with caution, as estimates may be subject to optimism.

Current trauma guidelines emphasize that imaging plays a central role in the evaluation of chest trauma, particularly in stable patients [10,26]. In the ABCDE flowchart, the Exposure may be sufficiently informative to confirm extra thoracic fractures or to elicit the suspicion of contralateral rib fractures. Extended focused assessment with sonography in trauma (eFAST) allows rapid identification of pneumothorax, pericardial effusion, and, fundamental for our experience, the presence of pleural effusion compatible with blood. As previously discussed, such a maneuver should not delay lifesaving interventions when tension physiology or hemodynamic instability is clinically evident [30], but in other cases, CTA is the diagnostic modality of choice for suspected thoracic vascular injury, with reported sensitivity approaching 95–100% and a negative predictive value of 99–100% [31,32,33].

Therefore, our findings support the role of early CTA evaluation in selected high-risk patients, particularly when these clinical predictors are present and the patient is sufficiently stable to undergo advanced imaging. In the near future, as is already done for acute stroke, we may envision CT scanners in ambulances, enabling immediate CTA evaluation at the trauma site.

This study has several limitations. Its retrospective design and single-center setting may limit generalizability, and imaging availability varied during the initial assessment. A preliminary power analysis was not performed given the retrospective design of the study. The highly selected nature of the study population, limited to patients with left-sided hemothorax admitted to a specialized thoracic unit, may have introduced selection bias and limited the generalizability of the findings. Additionally, hemothorax volume was assessed semi-quantitatively on chest radiography, reflecting real-world decision-making but lacking precise volumetric measurement. The absence of a significant temporal trend in the annual number of TEVAR procedures for BTAI supports the internal consistency of our findings despite potential changes in trauma management over time. Given the limited number of TEVAR events (*N* = 27), the multivariable model may be subject to overfitting, with potential overestimation of effect sizes and model performance. After internal validation using 5-fold cross-validation, the performance remained high (AUC 0.92; SD 0.04), with only a minimal reduction compared to the apparent estimate. This suggests limited optimism and good stability of the model despite the relatively small sample size and low number of events. The small difference between the apparent and cross-validated AUC indicates a low degree of overfitting, supporting the robustness of the selected predictors. However, these findings should be interpreted in the context of a retrospective single-center design and limited event rate, which may still affect generalizability.

Despite these limitations, the proposed model is simple, clinically applicable, and based on readily available parameters. It may assist in the early identification of patients at high risk of BTAI, supporting a CTA-first strategy and raising awareness regarding the potential risks of a drain-first approach.

## 5. Conclusions

BTAI requiring TEVAR is not uncommon in patients presenting with left-sided hemothorax after blunt thoracic trauma, even when pleural effusion is limited. High-energy trauma mechanism, extra-thoracic fractures, contralateral rib fractures, and low levels of hemoglobin at admission were identified as independent predictors of BTAI requiring TEVAR. In this selected cohort, these findings may support consideration of a CTA-first approach in hemodynamically stable patients at high risk of BTAI, when clinically feasible. In contrast, immediate chest drainage remains mandatory in the presence of life-threatening pleural conditions. These findings should not discourage urgent decompression when clinically indicated.

Overall, our results complement current trauma guidelines by identifying a subgroup of patients in whom early imaging may be prioritized to guide initial management. Further prospective validation is warranted to confirm these findings.

## Figures and Tables

**Table 1:** General data of the population.

Characteristic *	Data (*N* = 146)
**Age at trauma**	64 (50, 80)
**Gender**	
Female	40 (27%)
Male	106 (73%)
**Anticoagulant Therapy**	26 (19%)
**Antiaggregating Therapy**	13 (9.6%)
**INR**	1.10 (1.02, 1.27)
**Mechanism of Injury**	
High Energy	84 (59%)
Low Energy	58 (41%)
**Trauma Dynamic**	
Crash injury	66 (47%)
Fall	75 (53%)
**Fall distance (m)**	
1 m	52 (71%)
1 to 5 m	5 (6.8%)
over 5 m	16 (22%)
**Systolic BP at Admission (mmHg)**	138 (119, 150)
**Heart Rate at Admission (bpm)**	80 (72, 98)
**SaO2 at Admission (%)**	96 (94, 98)
**Temperature (°C)**	36.35 (36.00, 37.05)
**Shock status**	17 (12%)
**Prehospital IOT**	10 (7.2%)
**Hb at Admission (g/dL)**	13.40 (11.50, 14.70)
**Hct at Admission (%)**	41 (35, 43)
**Polytrauma CT immediately performed**	63 (43%)
**Traumatic Aortic Injury at the CT**	27 (18%)
**Site**	
Arch	3 (2.1%)
Complete	1 (0.7%)
Descending	6 (4.1%)
Isthmus	15 (10%)
Not reported	2 (1.4%)
No	119 (82%)
**Associated lesion to trauma**	
Dissection	8 (5.5%)
Not associated lesions	6 (4.1%)
Pseudoaneurysm	12 (8.2%)
Rupture	1 (0.7%)
No	120 (82%)
**Mediastinal Hematoma**	21 (14%)
**Rib fractures**	128 (88%)
**Number of rib fractures (RIGHT)**	0 (0, 0)
**Number of rib fractures (LEFT)**	4 (2, 6)
**Bilateral rib fractures**	18 (12%)
**Other thoracic fractures**	
Clavicula	20 (14%)
Clavicula, scapula	2 (1.4%)
Scapula	5 (3.4%)
Scapula, vertebra	2 (1.4%)
Sternum	7 (4.8%)
Sternum, vertebra	1 (0.7%)
Vertebra	8 (5.5%)
No fractures	101 (69%)
Extra thoracic fractures	39 (27%)
**Location of extra thoracic fractures**	
Arm	3 (2.1%)
Arm, leg, pelvis	1 (0.7%)
Arm, mandibula	1 (0.7%)
Arm, pelvis	3 (2.1%)
Arm, skull	1 (0.7%)
Leg	2 (1.4%)
Leg, pelvis, sacrum	1 (0.7%)
Leg, pelvis, vertebra	1 (0.7%)
Leg, skull	1 (0.7%)
Leg, vertebra	1 (0.7%)
Mandibula	2 (1.4%)
Mandibula, skull	1 (0.7%)
Pelvis	4 (2.8%)
Pelvis, sacrum, vertebra	1 (0.7%)
Pelvis, vertebra	2 (1.4%)
Skull	3 (2.1%)
Skull, pelvis	1 (0.7%)
Skull, vertebra	1 (0.7%)
Vertebra	10 (6.9%)
No fractures	105 (72%)
**Number of extra thoracic fractures sites**	
Multiple	18 (45%)
Single	22 (55%)
**Estimated effusion volume left hemithorax**	
High	4 (2.8%)
Intermediate	13 (9.0%)
Low	128 (88%)
**Left drainage placed**	32 (22%)
**Strategy**	
CT first and then directly in OR (BTAI)	4 (2.7%)
Drain after X-ray or CT scan	13 (8.8%)
Drain (for tension PNX, minimal effusion)	9 (6.2%)
Drain in the rescue field	6 (4.1%)
No drain	114 (78.1%)
**Later drainage**	
**Thoracentesis only**	2 (1.5%)
Yes	11 (8.0%)
No	124 (91%)
**Initial drained/estimated volume**	
500–1000 ml	16 (11%)
<500 ml	121 (85%)
>1000 ml	5 (3.5%)
**TEVAR required**	27 (18%)
**Bypass in addition to TEVAR**	
Carotid–Carotid	2 (1.4%)
Carotid–Subclavian	2 (1.4%)
Mesenteric artery stent	1 (0.7%)
**Other Surgery required**	14 (9.7%)
**Type surgery required**	
Leg amputation	1 (8.3%)
Thoracotomy/VATS and pleural toilette	10 (66.8%)
Splenectomy	1 (8.3%)
Wound exploration	1 (8.3%)
Subclavian stent	1 (8.3%)
**In hospital Mortality**	5 (3.5%)
30 days mortality	1 (0.7%)
90 days mortality	1 (0.7%)

Footnote: * Data are presented as Median and (IQR) or number (%).

**Table 2:** Comparative analysis between TEVAR group and no TEVAR.

Characteristic *	*N*	No TEVAR *N* = 119 (82%)	TEVAR *N* = 27 (18%)	*p*-Value
**Age at trauma**	104	68 (55, 82)	50 (31, 66)	0.002
**Gender (Males)**	146	86 (72%)	20 (74%)	0.85
**Anticoagulant Therapy**	136	23 (20%)	3 (14%)	0.57
**Antiaggregating Therapy**	136	11 (9.6%)	2 (9.1%)	>0.99
**INR**	64	1.06 (1.00, 1.23)	1.19 (1.05, 1.83)	0.076
**Mechanism of Injury (High Energy)**	142	60 (52%)	24 (89%)	<0.001
**Trauma Dynamic**	141			0.010
**Crash injury**		49 (43%)	19 (70%)	
**Fall**		65 (57%)	8 (30%)	
**Fall distance (m)**	73			<0.001
**1 m**		51 (78%)	1 (12%)	
**1 to 5 m**		4 (6.2%)	1 (12%)	
**over 5 m**		10 (15%)	6 (75%)	
**Systolic BP at Admission (mmHg)**	88	140 (124, 149)	120 (78, 155)	0.13
**Heart Rate at Admission (bpm)**	91	80 (71, 96)	91 (75, 107)	0.16
**SaO2 (%)**	83	96 (94, 98)	97 (93, 98)	0.68
**Temperature (°C)**	32	36.35 (36.00, 37.15)	36.30 (35.15, 37.00)	0.53
**Shock status**	139	3 (2.5%)	14 (70%)	<0.001
**Prehospital IOT**	138	3 (2.5%)	7 (37%)	<0.001
**Hb at Admission (g/dL)**	137	13.80 (12.05, 14.75)	10.95 (8.22, 13.78)	<0.001
**Hct at Admission (%)**	136	41 (37, 44)	34 (27, 41)	<0.001
**Polytrauma CT immediately performed**	145	38 (32%)	25 (96%)	<0.001
**Mediastinal Hematoma**	145	4 (3.4%)	17 (65%)	<0.001
**Rib fractures (*N*)**	146	110 (92%)	19 (70%)	0.004
**Number rib fract. (RIGHT)**	146	0 (0, 0)	0 (0, 4)	<0.001
**Number rib fract. (LEFT)**	146	4 (3, 6)	2 (0, 6)	0.042
**Bilateral rib fractures**	146	11 (9.2%)	7 (26%)	0.045

Footnote: * Data are presented as Median and (IQR) or number (%).

**Table 3:** Binomial logistic regression for TEVAR requirement.

Clinical Predictors	*p*	Odds Ratio	Lower	Upper
**Mechanism of Injury:**				
Low energy vs. High energy	0.048	5.080	1.0147	25.427
**Hb at Admission**	0.007	0.710	0.5536	0.911
**Number of rib fractures (RIGHT)**	0.018	1.432	1.0629	1.929
**Extra thoracic fractures**				
No vs. Yes	<0.001	12.922	3.5220	47.412

**Table 4:** Cross Table for radiological features requiring TEVAR after injury.

Characteristic *	*N*	No TEVAR *N* = 119 (82%)	TEVAR *N* = 27 (18%)	*p*-Value
**CT scan performed**	145	38 (32%)	25 (96%)	<0.001
**Mediastinal Hematoma**	145	4 (3.4%)	17 (65%)	<0.001
**Rib fractures**	146	110 (92%)	19 (70%)	0.004
**Number of rib fractures (Right)**	146	0 (0, 0)	0 (0, 4)	<0.001
**Number of rib fractures (Left)**	146	4 (3, 6)	2 (0, 6)	0.042
**Bilateral rib fractures**	146	11 (9.2%)	7 (26%)	0.045
**Extra thoracic fractures**	146	18 (14%)	22 (81%)	<0.001
**Estimated volume left hemothorax**	145			0.021
high		1 (0.8%)	3 (12%)	
intermediate		12 (10%)	1 (3.8%)	
low		106 (89%)	22 (85%)	
**Left drain**	146	24 (20%)	7 (26%)	0.51
**Strategy**	146			<0.001
Drain in the field		1 (0.8%)	5 (19%)	
Drain after Img		23 (19%)	2 (7.4%)	
No Drain		95 (80%)	20 (74%)	

Footnote: * *N* is the number of non-missing value. Pearson’s Chi-squared test; Fisher’s exact test; Wilcoxon rank sum test.

## Data Availability

The data presented in this study are available on request from the corresponding author. The data are not publicly available due to privacy and ethical restrictions.

## Abbreviations

The following abbreviations are used in this manuscript:

BTAI	Blunt thoracic aortic injury
TEVAR	Thoracic endovascular aortic repair
CTA	Computed tomography angiography
CT	Computed tomography
ED	Emergency Department
ICU	Intensive care unit
eFAST	Extended focused assessment with sonography in trauma
